# Development of a Measure to Assess What Young Heterosexual Adults Say They Learn About Sex from Pornography

**DOI:** 10.1007/s10508-021-02059-9

**Published:** 2021-11-10

**Authors:** Kate Dawson, Saoirse Nic Gabhainn, Malachi Willis, Pádraig MacNeela

**Affiliations:** 1 School of Psychology, National University of Ireland, Galway, Ireland; 2 School of Health Promotion, National University of Ireland, Galway, Ireland; 3 Social and Public Health Sciences Unit Institute of Health & Wellbeing, University of Glasgow, Glasgow, Scotland

**Keywords:** Pornography, Sexual information, Sex education, Porn literacy, Sexually explicit media

## Abstract

This study involved the development of two new measures to assess what some young heterosexual-identifying adults report learning about sex from pornography. Inventory items were generated from an extensive literature review and six qualitative focus group sessions with young adults (*N* = 54) aged 18–29 which explored how pornography could be used as a source of sexual information. A total of 135 items pertaining to sexual learning from pornography were produced, reviewed and categorized by a sample of young adults (*n* = 9), and finally reviewed for item and construct relevance by a panel of pornography, sex education and scale development experts (*n* = 6). Inventory items were administered to a sample of young adult university students (*n* = 1306). Two separate exploratory factor analyses were conducted for the female and male datasets. The final factors were reviewed by a panel of young adults  = 8) to identify the theme of each factor. Both the SIPI-F and SIPI-M yielded three factors: (1) How to be a good sexual partner, (2) Body aesthetic, and (3) Sexual exploration. Results show that pornography provides information about a range of sex-related topics. Findings also show that more frequent female pornography users reported learning more about how to be a good sexual partner and body aesthetic than less frequent pornography users. The SIPI-F and SIPI-M can be useful for examining a variety of questions regarding the use of pornography as an informal source of sexual information and its correlates.

**Supplementary Information:**

The online version contains supplementary material available at 10.1007/s10508-021-02059-9.

## Introduction

Watching pornography serves several functions, including sexual arousal, mood management, entertainment, curiosity, sexual exploration, and self-education (Bothe et al., 2020; Dawson, Cooper, et al., 2018; Dawson, Nic Gabhainn, et al., 2018; Grubbs et al., 2019; Paul & Shim, 2008; Smith et al., 2015; Wright, 2011). In terms of the latter function, young people feel motivated to engage with pornography to acquire information about sex (Aggarwal et al., 2000; Burns, 2001; Dawson, Nic Gabhainn, et al., 2019; Dawson, Tafro, et al., 2019; Doornwaard et al., 2017; Hald & Malamuth, 2008; McKenna et al., 2001; Rothman et al., 2015; Wright et al., 2018). However, there is a dearth of evidence about the educational value of pornography, specifically, what do young people report learning about sex from pornography? Some qualitative research indicates that, for young people, pornography may be considered a valuable source of information about sex because of its capacity to facilitate sexual experimentation, provide practical information about sexual acts, particularly anal and oral sex, and provide information regarding body function, genital aesthetic, sexual identity, and understanding of queer sexuality (Arrington-Sanders et al., 2015; Davis et al., 2017; Haggstrom-Nordin et al., 2006; Kubicek et al., 2010; Smith et al., 2015).

However, to date, no study has quantitatively catalogued the variety of information that a person reports learning from pornography nor the extent to which pornography contributes to their understanding of a particular concept. Therefore, this study aimed to bridge this gap by developing a measure to quantitatively assess what young Irish college students report learning about sex from pornography.

### Pornography Content

What a person learns about sex from watching pornography depends on the content they see (Wright, 2011). Pornography content varies greatly; one popular pornography content provider uploaded 4.8 million videos in 2018 alone (PornHub Insights, 2018). Content analyses of popular pornography have found that pornography portrays some common sexual behaviors, with vaginal sex, anal sex, fellatio, and masturbation being most frequently depicted (Carrotte et al., 2020; Downing et al., 2014; Vannier et al., 2014). Kissing, nipple stimulation, and manual anal penetration feature less often (Downing et al., 2014). Same-sex male pornography features condom use more often than pornography featuring couples of the opposite sex (Carrotte et al., 2020). Downing et al. (2014) found that approximately half of the gay anal sex scenes analyzed depicted condomless sex.

Mainstream pornography often depicts a standard body aesthetic; slim women with large breasts and men with larger than average penises (McKee et al., 2008; Schick et al., 2011). There are no content analyses of vulva appearance in mainstream pornography; however, one study previously identified a preference for hairless vulvas without protruding labia minora in popular pornographic magazines (Schick et al., 2011). It is worth noting that images in magazines may have been subjected to photo editing and may have not accurately portrayed the genitals of the models. Recent studies have also found disparities in the depiction of orgasm. Males orgasm more often in pornography than females orgasm (Klaasen & Peter, 2015; Seguin et al., 2018). For example, Seguin et al. (2018) found that 78% of popular mainstream pornography featured male orgasm, but only 18.3% depicted female orgasm.

Unequal sexual roles are also typical in mainstream pornography (Carrotte et al., 2020; Fritz & Paul, 2017; Klaassen & Peter, 2015). Men are depicted in dominant positions more often than women, and women are objectified more often than men (Klaassen & Peter, 2015). However, there are more representations of female sexual agency in queer feminist pornography (Fritz & Paul, 2017). Fritz and Paul (2017) also found that female objectification was more common in mainstream pornography than in feminist and “for women” pornography. Women are also more often depicted as the targets of aggression in pornography (Carrotte et al., 2020).

Carrotte et al. (2020) and Klaassen and Peter (2015) found that, beyond portraying acts of aggression, like spanking and gagging, pornography rarely depicted acts of extreme violence and rape. In their analysis of 210 popular Pornhub videos, Seida and Shor (2019) found that same-sex content depicted more acts of aggression and more displays of affection than opposite sex videos. Fritz et al. (2020) found that aggression features more often in pornography scenes that featured black couples. They also highlighted that black women are targets of sexual aggression more often than white women and that black men perpetrate more acts of aggression than other men (Fritz et al., 2020). Willis et al. (2020) found that pornography depicts non-verbal cues more often than verbal cues and depicts women as indirect and men as direct communicators of consent. The study also found that pornography seldom depicted explicit consent for lower-order behaviors and that, in some scenarios, a performer could consent by doing nothing (Willis et al., 2020).

Individual characteristics impact the content someone sees and how it is perceived (Davis et al., 2018). A recent study by Davis et al. (2018) highlights how young heterosexual adults differ in the frequency with which they see various behaviors in mainstream pornography. While both men and women report regularly seeing depictions of male dominance and pleasure, women were more likely to report seeing violence against women than men, and men reported seeing more anal sex than women.

### What do People Learn About Sex from Watching Pornography?

Individuals may intentionally seek out pornography to learn about sex, while others may incidentally learn through watching pornography. Wright’s 3AM model (2011) posits that several audience and content factors play a role in how pornography influences personal sexual learning. Audience factors, such as individual motivation for use and content factors, including the perceived utility of pornography, are essential in this context.

Several studies show that young people report using pornography as a source of sexual information (Aggarwal et al., 2000; Doornwaard et al., 2017; Rothman et al., 2015; Wright et al., 2018). Studies show that youth often use pornography as a source of information about sexual practices like oral and anal sex (Duncan & Donnelly, 1991; Haggstrom-Nordin et al., 2006; Kubicek et al., 2010). Other studies show that young people report using pornography to learn about sexual techniques and performance (Arrington-Sanders et al., 2015). Using pornography as a source of sexual health information has been associated with taking more sexual risks (Rosengard et al., 2012).

Studies have shown that those who rate pornography as their primary educator about sex are less likely to use condoms (Wright et al., 2018, 2019). Some youth report using pornography to learn about sex in the absence of good quality sex education (Arrington-Sanders et al., 2015). However, one recent study also found no significant difference between those who were satisfied with the school-based sex education and those who were not regarding their use of pornography as an informal source of sex education—indicating that young people use pornography to learn about sex regardless of the quality of sex education that they receive (Dawson, Cooper, et al., 2018; Dawson, Nic Gabhainn, et al., 2018). However, there is little consensus among scholars and educators on what pornography teaches its consumers (Albury, 2014).

Although not originally designed to act as an educator, some pornography includes educational elements that focus on enhancing sexual pleasure and facilitating safer sex practices. Early examples of this include “Nina Hartley’s guide to better cunnilingus” which provides practical steps for couples to improve or enhance female sexual pleasure through oral sex (Hartley, 1995). More recently, CrashPad series by Pink and White Productions (2016) produced content that portrays sexual negotiation strategies and the use of safewords as part of Bondage, Dominance, and Sadomasochism (BDSM) practices. Educational value is not an essential component of the majority of mainstream pornography. However, for some, it has become an informal educational resource (Aggarwal et al., 2000; Doornwaard et al., 2017; Rothman et al., 2015; Wright et al., 2018).

Recent results from a systematic review of the qualitative and quantitative literature show that young peoples’ reported learning from pornography can be summarized as learning about the mechanics of sex, including sexual techniques and fetish or kink behaviors, and about sexuality and sexual identities, including one’s own sexual identity and that of others (Litsou et al., 2020). However, the study also found that young people report that much of the information they obtain from pornography is inadequate or unreliable (Litsou et al., 2020). For example, several studies report conflicting findings regarding youth perceptions about pornography for sexual information. For example, some believe mainstream Internet pornography to be a valuable source of information for some queer youth in exploring their sexuality and has helped some same-sex attracted people to increase their sexual confidence (Arrington-Sanders et al., 2015). In contrast, Dawson, Nic Gabhainn, et al. (2019), Dawson, Tafro, et al. (2019)) highlighted how some youth challenge the representation of LGBT + people in pornography for portraying negative stereotypes about queer-identifying people. Similarly, recent findings show that pornography provides positive learning opportunities for some young women but not others. Davis and colleagues (2017) found that for some individuals, the opportunity to explore genital aesthetics by watching pornography was a liberating experience that broadened their understanding of the female body, while others reported a negative impact concerning their own and their partner's sexual expectations for genital function and aesthetic (Davis et al., 2017). Much of the research in this area has focused on young women’s and LGBT + persons’ learning. Young men report using pornography for information more often than young women (Tanton et al., 2015), yet a dearth of research highlights what information young men learn in this context.

### The Current Study

While some have found that individuals actively use pornography to learn about sex, learning may also occur without engaging with pornography in order to learn (Tanton et al., 2015; Wright et al., 2018, 2019). In addition, qualitative studies compose much of the literature in this area. To date, quantitative studies have not addressed the range of learning that can take place from engaging with pornography. This study involves developing a new measure to quantitatively catalogue the range of learning that some young Irish college students report from engaging with pornography. This study also assesses the differences in reported learning between less frequent and more frequent pornography users. The study received full approval from the Research Ethics Committee at the X University.

## Method

### Recruitment

Qualitative data (*n* = 54) were collected at X university. For more information regarding recruitment for Phase 1, please see (blinded for review). We collected the quantitative data at two third-level education institutions in Ireland; a university and a technical institute. Invitations to participate were sent to a mixed-gender, convenience sample of registered students aged 18 + via an anonymous online survey. In total, 17,000 students received e-mail invitations via the internal student e-mail system at each institution. In total, 1306 heterosexual students took part in the questionnaire. Data collection took place in September 2018.

#### Inclusion Criteria

We informed participants of the questionnaire's explicit nature. All students received a detailed study overview before participating. We invited those who gave their informed consent and were over the age of 18 to participate. We chose a mixed-gender sample of 18–29-year-olds for a number of reasons. Research suggests some gender and sexual orientation-specific differences in the use of pornography for sexual information. For example, women report learning about female genital aesthetics from pornography (Davis et al., 2017), and same-sex attracted youth often report using pornography as a means to explore their sexual orientation (Arrington-Sanders et al., 2015). Therefore, we sought to recruit a large and varied sample, which would allow us to explore the factor structure with different groups.

We received 1306 responses from heterosexual-identifying young adults. A total of 115 LGBT + -identifying young adults also responded to the questionnaire. However, the sample size limited our ability to conduct meaningful factor analyses with the LGBT + dataset. The current study remains ongoing and will employ targeted sampling to recruit a larger sample of LGBT + young adults. We conducted the final analysis with heterosexual-identifying young adults. The majority of participants were Irish (77%) and were 18–21 years of age (68%). For detailed participant information, see Table 1.

**Table 1:** Participant demographic information *n* (%)

	Female	Male	Total
*Age*			
18–21	515 (75)	413 (66.5)	928 (71)
22–25	129 (19)	149 (24)	278 (21)
26–29	41 (6)	59 (9.5)	100 (80)
*Relationship status*			
Not in a relationship	298 (43.5)	338 (54)	636 (49)
Casual dating	76 (11)	48 (8)	124 (9.5)
Dating a partner and others	8 (1)	14 (2)	22 (2)
In a relationship but not living with partner	250 (36.5)	181 (29)	431 (33)
In a relationship and living with partner	53 (8)	40 (6)	93 (7)
*Nationality*			
Irish	529 (77.5)	476 (77)	1005 (77)
Non-Irish	156 (22.5)	143 (23)	297 (23)
*Are you religious?*			
Yes	240 (35)	169 (28)	409 (32)
No	438 (65)	442 (72)	880 (68)
Total	685	621	1306

### Measures

#### Defining Pornography

We defined Internet pornography as “Website content that has descriptions, pictures, movies, or audio of people having sex or engaging in other sexual behaviors,” based on recommendations by Kohut (2014) about young people’s definitions of internet pornography.

#### Sexual Information from Pornography Inventory (SIPI)

We asked participants to read the following question: “Please indicate the degree to which you have learned something about the following items from watching porn.” Responses were recorded on a 5 point Likert-style scale (1. Nothing, 2. A little, 3. Some, 4. A lot, 5. Everything). Each participant received a randomized set of the complete scale item list. See the Appendix for the original item list.

#### Frequency of Pornography Engagement

We measured participants frequency of pornography engagement on a five-point Likert scale (Hald, 2006) (Never; Less than once a month; 1–2 times per month; 1–2 times per week; 3 times per week or more). For the purpose of analysis, less frequent (Less than once a month, 1–2 times per month) and more frequent pornography (1–2 times per week, three times per week or more) users were combined to create a binary variable.

### Procedure

#### Phase 1: Group Discussion Qualitative Analysis and Literature Review

Group discussions generated relevant themes that provided the basis for an extensive review of the pornography research literature according to the themes identified. There were 54 participants whose ages ranged from 18 to 29. The group discussions sought to explore (1) the beliefs that young adults had about the messages that pornography promoted, (2) what young people learn about sex from watching pornography, and (3) the core messages for pornography education for young people. We then used the descriptive themes from the group discussion data in the development of the survey items. For full information regarding the development of themes, please see (blinded for review). We translated each theme into multiple Likert-type questions, which represented core aspects of that theme. For example, one recurring theme related to the belief that pornography promoted unattainable body image standards for men and women, and the theme of body image was elaborated upon to develop neutral items such as “what naked bodies look like,” “what breasts look like,” and “what male genitals look like.” We generated a total of 160 items from this phase. For detailed information on participants and on theme development, please see: (blinded for review).

#### Phase 2: Item Revision, Expansion and Amendment

We then invited a small panel of young adults (*n* = 9), who had each attended one of the group discussions, to review the item pool. Participants reviewed the groups of items and their related themes and worked in small groups of 3 people to:

1. Identify overlapping questions; in doing this, we asked participants to retain the item which best conveyed the item at hand and to discard the others. If a pre-existing item was deemed unclear, participants could re-write the question using language that was most accessible to them.
2. Expand on the item list to provide more detailed items related to a broader theme. For example, participants included the item “About squirting/vaginal ejaculation,” as they believed that the item related to learning about female genital functioning was too broad.
3. Review the items for clarity and to rephrase where appropriate,
4. Recommend items which, through group consensus, emerged to be unnecessary or irrelevant.
5. Include new items that they believed would provide essential additional information to the inventory. A total of seventeen items, which pertained to watching pornography to learn about other people’s perceptions, for example, “to see what types of bodies other people think are attractive,” were removed, leaving 135 items.

Face validity. We invited six experts in pornography research, sex education, and scale development to rate the items’ clarity, relevance to the research, and comprehensiveness. Experts (1) rated how relevant they think each item is to what we are measuring, i.e., learning from pornography, using a 4 point scale (1 = not relevant, 2 = somewhat relevant, 3 = quite relevant, 4 = highly relevant), (2) generated new items if they found some aspect missing, (3) improved items to better capture the concept, (4) provided comment on item clarity and conciseness and (5) provided a rationale if they recommended discarding an item entirely.

#### Phase 3: Pilot testings to Ensure Item Clarity and Meaningfulness

The final stage in developing the item list involved administering the 137 item inventory to a sample of 14 university students to assess for suitable readability. Participants rated all items as clear. Some participants reported that some items overlapped, i.e., the items explored the same concept, for example, ‘About sexual behaviors heterosexual people engage in’ and ‘About heterosexual sexual behaviors.’ Therefore, participants reported which overlapping items provided the most concise description and were most relevant to the inventory to reduce the number of items to a more meaningful set.

#### Phase 4: Data Analysis

We first analyzed the full recruited sample of heterosexual and LGBT + young adults (*n* = 1461). This produced no meaningful factor structure and eliminated items of importance for specific groups. For example, among the LGBT + cohort, mean scores related to learning about same-sex sexuality produced high mean scores. However, these items did not load onto any factor when included with the larger heterosexual cohort analysis. We decided to focus the current analysis on heterosexual young adults and continue data collection at a later stage among LGBT + young adults to produce a more meaningful set of factors for different orientation groups.

The first stage of data analysis with the heterosexual sample involved removing items with a mean value below 1.5, indicating that the sample reported learning nothing about it from watching pornography.

### Exploratory Factor Analysis

We randomized and split the female and male datasets to produce an exploratory factor analysis (EFA) and confirmatory factor analysis (CFA) dataset for each gender group. We conducted EFAs using a direct oblimin rotation and maximum likelihood as a method of extraction on the SIPI datasets to explore item groupings and to produce a set of items to retain in the final measures. Decisions regarding factor retention were made based on the variance explained, eigenvalues above 1, scree plot illustrations, as well as conceptual considerations for item relevance to each factor (Osborne & Costello, 2004; Worthington & Whittaker, 2006). We assigned a label to each factor that reflected what each factor represented. We used SPSS version 22 for analysis. We conducted two separate analyses to explore the factor structure among women and men. We report reliability analysis of the full SIPI-F and SIPI-M scale and their subscales using Cronbach’s alpha coefficients.

We conducted Kaiser–Meyer–Olkin tests of sampling adequacy (KMO) to determine whether male and female samples were sufficiently large to conduct factor analysis (Osborne & Costello, 2004). We used Maximum likelihood (ML) as the method for data extraction. Correlations between factors were assumed, and therefore an oblique rotation with Kaiser normalization was used (Byrne, 2016). Theory, scree plot illustrations and eigenvalues determined the number of factors. Regarding factor loadings, Howard (2016) recommended that satisfactory items should load onto their primary factor above 0.40, load onto alternative factors below 0.30 and exhibit a difference of at least 0.20 between the primary factor and other factors. The current article follows this recommendation.

### Confirmatory Factor Analysis

We conducted CFAs of the three factors generated for both female and male measures during EFA on our confirmatory datasets using AMOS 24 (Arbuckle, 2011). We used ML as a method of extraction. An a priori decision was made to retain any items with loadings greater than 0.30 (Byrne, 2016). Regarding the model fit, we chose a priori to interpret the comparative fit index (CFI), a practical model fitting criterion that also considers the sample size and the Tucker-Lewis index (TLI). CFI and TLI values close to 0.95 represent a good model fit. We interpret the root mean square error of approximation (RMSEA) because of its sensitivity to model misspecification, model quality and its capacity to produce confidence intervals, which allow the researcher to assess the precision of the estimates produced (Byrne, 2016). Recommended RMSEA values should be less than 0.06 (Byrne, 2016). Finally, we interpret the standardized root mean square residual (SRMR), which should be less than 0.08 to indicate good model fit.

Because all items used five-point rating scales and may thus be considered categorical, we used a robust diagonally weighted least squares (DWLS) estimator for the CFAs. The DWLS estimation technique is recommended for data that are nonnormally distributed or categorical in nature (Muthén & Muthén, 2009).

### Subtheme Identification

The first author and a group of young adults (*n* = 8) reviewed the final item lists to identify the underlying theme and its corresponding heading for each factor (Table 2).

**Table 2:** Frequency of pornography engagement *n* (%)

Frequency	Female	Male	Total
3 times per week or more	9 (1)	145 (23.5)	154 (12)
1–2 times per week	61 (9)	299 (48.5)	360 (28)
1–2 times per month	182 (27)	130 (21)	312 (24)
Less than once a month	222 (33)	31 (5)	253 (19.5)
Never	208 (30.5)	11 (2)	219 (17)
Total	682 (100)	616 (100)	1298 (100)

## Results

### Exploratory Factor Analysis (SIPI-F)

The KMO result from the EFA female-only dataset (*n* = 339) was 0.826, suggesting factor analysis was appropriate for use on our data set. Anti-image matrices showed that partial correlations between variables were small, with diagonals ranging between 0.378 and 0.749. EFA produced three factors that explained 51.03% of the variance. Factor loadings ranged from (0.442) to (0.845). Factor 1 contains seven items; Factor 2 contained nine items; Factor 3 contained three items. See Table 3 for factor scores.

**Table 3:** EFA factor loadings (*F*), mean and standard deviations (SD) for the sexual information from pornography inventory-female (SIPI-F*)*

Subtheme	F1	F2	F3	M	SD
*How to be a good sexual partner*					
How to be good in bed	**.821**	.139	− .235	2.46	1.22
What women should do during sex	**.782**	− .246	.200	2.56	1.30
About oral sex on male genitals	**.762**	.130	− .136	3.20	1.30
Performing hand jobs	**.684**	.035	.066	2.79	1.32
About male erections	**.673**	− .016	.026	2.76	1.37
How to give pleasure to a sexual partner	**.632**	.049	.106	2.81	1.24
About male orgasms	**.551**	− .022	.170	2.70	1.35
Total				2.75	
*Sexual exploration*					
About dominance and/or submission	− .058	**.707**	− .021	2.90	1.43
About things I didn’t know would turn me on	− .087	**.707**	.152	2.86	1.31
About different sexual fantasies	.048	**.698**	.005	2.74	1.34
About role playing	.166	**.589**	− .013	2.48	1.80
About bondage	.109	**.572**	− .117	2.88	1.80
About sadomasochism	− .106	**.536**	− .078	3.40	2.23
My own sexual boundaries	− .034	**.502**	.284	2.94	1.41
About things I’d like to try with my partner	.261	**.490**	.058	2.96	1.21
About foreplay	.221	**.442**	.140	2.77	1.38
Total				2.88	
*Body aesthetic*					
What naked bodies look like	.085	− .092	**.845**	3.16	1.28
What breasts look like	− .143	.104	**.742**	2.79	1.47
What genitals look like	.298	− .067	**.579**	3.16	1.36
Total				3.04	

Items in bold load to corresponding factor

### Confirmatory Factor Analysis (SIPI-F)

To test the proposed three-factor structure based on the EFA results for the SIPI-F, we then conducted a CFA using the separate female-only dataset (*n* = 346), *χ*^2^(149) = 188.29, *p* = 0.016, CFI = 0.916, TLI = 0.904, RMSEA = 0.046, SRMR = 0.058. Even though the *χ*^2^ test statistic was significant, the alternative fit indices were close to recommended cut-offs and, thus, suggested that the model adequately fit the data.

### Exploratory Factor Analysis (SIPI-M)

The KMO measure for the male-only dataset (*n* = 340) was 0.617, suggesting factor analysis was adequate for use on our data set. Anti-image matrices showed that partial correlations between variables were small, with diagonals ranging between 0.182 and 0.648. EFA resulted in three factors, which explained 54.27% of the variance. Factor loadings ranged from (0.533) to (0.891). Factor 1 contains 16 items; Factor 2 contained six items; Factor 3 contained four items. See Table 4 for factor scores.

**Table 4:** EFA factor loadings (F), mean and standard deviations (SD) for the sexual information from pornography inventory-male (SIPI-M)

Subtheme	F1	F2	F3	Mean	SD
*How to be a good sexual partner*					
About what good sex is like	**.891**	.025	− .178	2.39	1.19
How to communicate non-verbally during sex	**.882**	.020	− .076	2.29	1.21
How to give pleasure to a sexual partner	**.861**	− .190	− .008	2.83	1.09
How to interact with a partner during sex	**.816**	.027	.026	2.40	1.13
How to be good in bed	**.773**	.046	− .143	2.32	1.17
How to read a sexual partner’s body language	**.749**	− .102	− .059	2.06	1.21
About female orgasms	**.736**	.134	− .053	2.61	1.13
About what is expected of a person when having sex	**.712**	− .011	.047	2.54	1.18
How to communicate verbally during sex	**.701**	− .007	.032	2.20	1.16
How to ‘talk dirty’	**.670**	.006	− .062	2.65	1.20
How to achieve mutual pleasure	**.657**	.004	.102	2.26	1.03
What men should do during sex	**.632**	− .037	.281	2.51	1.19
About things I’d like to try with my partner	**.592**	− .028	.024	3.24	1.16
How to make a sexual partner have an orgasm	**.574**	.092	.248	2.41	1.21
What I would feel comfortable doing in bed	**.545**	.037	.143	2.90	1.09
How to make a partner ‘squirt’	**.533**	.114	.158	2.06	1.17
Total				2.45	
*Sexual exploration*					
Penetration of anus by finger(s)	.086	**.825**	.063	2.73	1.54
About anal fisting	.073	**.800**	− .048	2.53	1.84
How to have group sex	.073	**.763**	− .159	2.57	1.71
About vaginal fisting	.022	**.579**	.014	2.45	1.67
About strap-on intercourse	.067	**.559**	.019	2.74	1.72
About anal sex	.157	**.509**	.121	2.88	1.29
Total				2.65	
*Body aesthetic*					
What naked bodies look like	.111	− .100	**.890**	3.38	1.18
What genitals look like	.065	.073	**.783**	3.03	1.16
What breasts look like	.048	− .071	**.763**	3.21	1.20
About vulva appearance (what the outside of the vagina looks like)	.104	.098	**.633**	2.94	1.20
Total				3.14	

Items in bold load to corresponding factor

### Confirmatory Factor Analysis (SIPI-M)

To test the proposed three-factor structure based on the EFA results for the SIPI-M, we then conducted a CFA using the separate male-only dataset (*n* = 281), *χ*^2^(296) = 326.68, *p* = 0.106, CFI = 0.949, TLI = 0.944, RMSEA = 0.032, SRMR = 0.068. The non-significant *χ*^*2*^ test statistic indicated that the model was an adequate fit for the data; this was further supported by the alternative fit indices.

### Reliability

Internal consistency was demonstrated for the SIPI-F overall (*α* = 0.94) and for its individual subscales: Subscale 1 (*α* = 0.90), Subscale 2 (*α* = 0.88), Subscale 3 (*α* = 0.82).

Internal consistency was demonstrated for the SIPI-M overall (*α* = .93) and for its individual subscales: Subscale 1 (*α* = 0.94), Subscale 2 (*α* = 0.84) and Subscale 3 (*α* = 0.86).

### Frequency of Pornography use and Reported Learning

We conducted a series of independent samples T-tests to explore the reported learning from pornography for less frequent and more frequent pornography users.

#### Women

A statistically significant difference between less frequent and more frequent female viewers was also observed regarding subscale 1, being a good sexual partner *t*(183) = 3.10, *p* = 0.002 and subscale 3, body aesthetic *t*(179) = 2.23, *p* = 0.027. More frequent viewers (*M* = 20.03, SD = 7.79) reported learning more about being a good sexual partner from watching pornography than less frequent viewers (*M* = 15.81, SD = 6.41). More frequent female viewers reported greater learning (*M* = 21.54, SD = 12.75) regarding body aesthetics than less frequent viewers (*M* = 18.26, SD = 11.43).

#### Men

There was no significant difference between less (26.17, SD = 15.30) and more (*M* = 29.42, SD = 16.28) frequent male viewers regarding their reported learning for Subscale 1 *t*(211) = 1.28, *p* = 0.203. There was no significant difference between less (*M* = 12.39, SD = 7.49) and more (*M* = 14.89, SD = 7.96) frequent viewers for Subscale 2 *t*(171) = 1.84, *p* = 0.067. Nor was there a significant difference between less (*M* = 10.75, SD = 4.24) and more (M = 11.10, SD = 4.46) frequent viewers for Subscale 3 *t*(189) = 0.473, *p* = 0.637.

## Discussion

The current study sought to quantitatively assess what young Irish college students report learning about sex from watching pornography. This research is important given the growing body of literature, which shows that many young people use pornography as an informal educational resource (Dawson, Cooper, et al., 2018; Dawson, Nic Gabhainn, et al., 2018; Litsou et al., 2020; Tanton et al., 2015; Wright et al., 2018, 2019). This study identified several aspects of perceived learning from pornography. The current study did not measure objective learning from pornography. Therefore, findings may speak to what students seek to learn from pornography rather than their actual learning. There may be significant discrepancies between what young adults try to learn or think they are learning versus what knowledge they gain. We did not assess students' perceptions regarding the value of the information they reported acquiring from pornography. Several studies have highlighted that some young people report learning inaccurate and unreliable information about sex from pornography, while others report acquiring helpful information (Arrington-Sanders et al., 2015; Davis et al., 2017; Dawson, Nic Gabhainn, et al., 2019; Dawson, Tafro, et al., 2019; Litsou et al., 2020).

Women and men reported learning about similar themes from watching pornography. However, the inventory items which corresponded to their reported learning differed regarding their respective contexts. For heterosexual women being a good sexual partner involved pleasing a male partner and performing manual and oral male genital stimulation. For heterosexual men, being a good sexual partner concerned how men should behave during sex and how to please their female partner. Regarding sexual exploration, the SIPI-F inventory included more varied practices, including those related to BDSM. In practice, the prevalence of BDSM practices is higher among men than women (Neef et al., 2019). Our findings may therefore indicate that young women have more varied interests when it comes to exploring their sexuality online but may be less likely than young men to participate in the behaviors they see in pornography. For the young men in our sample, sexual exploration involved a focus on a wider variety of penetrative sexual practices. The findings indicate that, in this context, in terms of using pornography for sexual exploration, some young heterosexual men and women differ. For women, learning about role playing, sexual fantasies and BDSM practices suggests that sexual exploration relates to expanding their knowledge about a range of experiences, not only sexual acts. For men, these experiences focus on a greater extent on sexual acts, specifically involving penetration.

Among a cisgender and heterosexual college sample, our findings show some items, in particular, showed higher mean scores, including “what naked bodies look like,” “what genitals look like” and “different sexual positions.” Mean scores were higher for women who reported engaging with pornography more frequently. How accurate or reliable the knowledge obtained from pornography is uncertain. Some argue that pornography can provide reliable information regarding sexual practices and body parts (McKee, 2010). Detailed depictions of varied genitalia and the range of sexual behaviors are usually not part of school-based sexual health programs in Ireland (Bewiser, 2019; Foroige, 2019). Pornography may, therefore, potentially provide valuable information for young people. However, we do not know if learning from pornography contributes to an improvement in one’s sexual knowledge (Litsou et al., 2020). Future studies should explore if the acquisition of information from pornography is associated with such improvements.

Considering youth often report that their school-based sex education leaves out important information that they want, pornography may fill this knowledge gap concerning sexual behavior, body aesthetics and sexual exploration. Although some pornography may provide accurate depictions of these concepts, the variability in pornography means that it is unlikely that all depictions will provide accurate or reliable information. Pornography may set sexual norms about behavior and functioning, particularly if there is no available information about the realities of some practices. This is particularly problematic for youth who may choose to engage with non-consensual and violent pornography. Although many young people may see non-consensual depictions in pornography, for example, Davis et al. (2018), in their study with 517 Australian young people, found that 18% reported seeing pornography content that depicted non-consensual violence toward a man, and 43.5% reported seeing non-consensual violence toward a woman, in the past 12 months. However, much smaller percentages show a preference for coercive and violent content. Landripet et al. (2019) found that only 5–8% of male Croatian adolescents preferred aggressive pornographic content. Several studies have identified preferences for violent pornography as a predictor for sexual violence (Dawson, Nic Gabhainn, et al., 2019; Dawson, Tafro, et al., 2019; Wright et al., 2015; Ybarra & Thompson, 2018).

Improving sex education, which focuses on the identified factors, is therefore essential. Sex education, which discusses pornography, the realities of sex, realistic depictions of a variety of genital and body types, and which focuses on sexual pleasure, are paramount to the sexual wellbeing of young people (Albury, 2014; Bengry-Howell, 2012; Dawson, Nic Gabhainn, et al., 2018; Dawson, Cooper, et al., 2018; McKee et al., 2010). However, such programs are not widely accessible, and many are based on policy guidelines and recommend educational initiatives that focus predominantly on sexual risks (Mayock et al., 2007). Therefore, the findings may help sex educators and those who develop sexual health interventions by highlighting the critical areas for improvement in sex education programs. Providing information about real-world sexual experiences and allowing youth time to explore these topics in individual and group settings may reduce the need for young people to use pornography as an educational resource.

### Limitations and Future Research

The current study has several limitations that warrant discussion. Our convenience sample of young, Irish university students is not representative of the rest of the population. We assessed students' nationality, but not their race or ethnicity. Data show that 15% of Irish college students identify with a non-white Irish ethnic group (Higher Education Authority, 2018), but we do not know how many identified as non-white Irish students in our sample. Indeed, religious and cultural backgrounds may reduce the likelihood that certain groups participate in studies about sexuality (Strassberg & Lowe, 1995). The heterosexual-identified sample of university students limits the measure's reliability among queer-identifying youth and other age cohorts (Henrich et al., 2010). A significant proportion of survey respondents was queer-identifying. However, we did not have a large enough sample of queer-identifying youth to conduct robust analyses. We invite researchers to replicate this study to explore the factor structure among more varied samples. Additionally, as there was no similar measure to the SIPI-F and SIPI-M, we could not assess concurrent validity.

Future studies should explore the level of sexual experience and sexual knowledge obtained from parents or school-based sex education. Those who have a great deal more sexual experience or who had good quality sex education may differ in their extent of reported learning about sex from pornography. The proportion of information that a person has learned about a sexual topic from watching porn is likely to be reduced or exaggerated, depending on the level of information about sex and direct sexual experience that they have. In addition, participants were asked to report the degree of learning about each item from watching pornography; however, as with all self-reported data, relying on subjective responses, participants may have interpreted the response options differently. In the current study, we were interested in internet pornography and used a rather “strict” definition of pornography. We did not consider pornographic magazines and other non-Internet-based sexual material for the current analysis. Individuals who read pornographic magazines or erotic novels that some would consider pornographic may also be educational, particularly in terms of learning about sexual body parts and providing a space for sexual exploration.

### Implications for Future Research

Future research should assess the type of pornography content with which an individual engages. Those who watch a greater variety of videos may have learned more than those who habitually engage with similar content. Future research could explore whether participants with a more complex range of content choices learn more about sex from pornography. Although participants reported learning about sex from pornography to different extents, it was beyond this study's remit to explore whether such learning influences their behavior or feelings about themselves. The development of this measure allows for a more thorough exploration of such associations in future research. Future studies should explore whether those who actively engage with pornography for information report greater learning from pornography as a result. Actively engaging with pornography for sex information, instead of passive viewing, may be associated with more substantial social comparison effects if one purposefully uses a media source to gain information about behavior. Finally, what a person learns about sex from pornography may be influenced by their gender, sexual orientation, motivation for engagement among a series of other personal and cultural characteristics, which influence the reception of and reaction to pornography (Wright, 2011). Although individuals may see the same content, it can be interpreted differently depending on a person's attitude toward pornography, beliefs about its effect, sexual self-esteem and several additional factors.

### Conclusion

The SIPI-F and SIPI-M presented here can help to advance our understanding of the role of pornography as an informal source of sexual information. The findings demonstrate that young people may find pornography useful regarding knowledge acquisition related to a specific number of constructs, including sexual behavior, sexual exploration and body aesthetics. Future research should test the construct validity of this measure across diverse samples of young adults, including different ethnic groups, large samples of lesbian, gay and bisexual young people, as well as transgender and gender non-conforming individuals.

### Supplementary Information

Below is the link to the electronic supplementary material.

Supplementary file1 (DOCX 23 kb)

## Appendix

See Tables

**Table 5:** CFA factor loadings for the SIPI-F

	Standardized loading	Reliability (*α*)
Subscale 1: how to be a good sexual partner		.90
How to be good in bed	.827	
What women should do during sex	.730	
About oral sex on male genitals	.798	
Performing hand jobs	.787	
About male erections	.712	
How to give pleasure to a sexual partner	.832	
About male orgasms	.722	
Subscale 2: sexual exploration		.88
About dominance and/or submission	.812	
About things I didn’t know would turn me on	.666	
About different sexual fantasies	.781	
About role playing	.731	
About bondage	.669	
About sadomasochism	.428	
My own sexual boundaries	.662	
About things I’d like to try with my partner	.778	
About foreplay	.823	
Subscale 3: body aesthetic		.82
What naked bodies look like	.846	
What breasts look like	.714	
What genitals look like	.869	

^5^ and

**Table 6:** CFA factor loadings for the SIPI-M

	Standardized loading	Reliability (*α*)
Subscale 1: how to be a good sexual partner		.94
About what good sex is like	.815	
How to communicate non-verbally during sex	.634	
How to give pleasure to a sexual partner	.825	
How to interact with a partner during sex	.800	
How to be good in bed	.708	
How to read a sexual partner’s body language	.617	
About female orgasms	.785	
About what is expected of a person when having sex	.829	
How to communicate verbally during sex	.640	
How to ‘talk dirty’	.783	
How to achieve mutual pleasure	.714	
What men should do during sex	.851	
About things I’d like to try with my partner	.610	
How to make a sexual partner have an orgasm	.815	
What I would feel comfortable doing in bed	.693	
How to make a partner ‘squirt’	.498	
Subscale 2: sexual exploration		.84
Penetration of anus by finger(s)	.642	
About anal fisting	.655	
How to have group sex	.608	
About vaginal fisting	.689	
About strap-on intercourse	.595	
About anal sex	.901	
Subscale 3: body aesthetic		.86
What naked bodies look like	.781	
What genitals look like	.757	
What breasts look like	.752	
About vulva appearance (what the outside of the vagina looks like)	.724	

^6^. See supplementary material for full list of original inventory items.
