# Supplementary material for: Development of a Measure to Assess What Young Heterosexual Adults Say They Learn About Sex from Pornography
Source: Arch Sex Behav. 2021 Nov 10;51(2):1257–69. doi: 10.1007/s10508-021-02059-9 (PMC8888499; doi:10.1007/s10508-021-02059-9)
Supplement: Supplementary file 1 — Supplementary file1 (DOCX 23 kb) [file 10508_2021_2059_MOESM1_ESM.docx]

| **Table 7.** *Original Inventory Items Retained for SIPI-F and SIPI-M* (✓) |  |  |
| --- | --- | --- |
| Original Inventory Items | SIPI-F | SIPI-M |
| Performing oral sex on female genitals |  |  |
| How to communicate verbally during sex |  | ✓ |
| About same sex sexual positions |  |  |
| The places where people have sex |  |  |
| About male orgasms | ✓ |  |
| About sexual fetishes |  |  |
| What naked bodies look like | ✓ | ✓ |
| How to give pleasure to a sexual partner | ✓ | ✓ |
| About fetish communities |  |  |
| Performing hand jobs | ✓ |  |
| To see what things other people watch |  |  |
| How to fake sexual pleasure |  |  |
| How intersex persons behave during sex |  |  |
| About the use of dental dams |  |  |
| What bodies are attractive to other people |  |  |
| About male erections | ✓ |  |
| About clitoral erections |  |  |
| What naked transgender persons’ bodies look like |  |  |
| About popular sexual behaviours |  |  |
| About things I’d like to try with my partner | ✓ | ✓ |
| How many orgasms a person can have |  |  |
| What I would feel comfortable doing in bed |  | ✓ |
| How to get into different sexual positions |  |  |
| Squirting/vaginal ejaculation |  |  |
| How men behave during sex |  |  |
| What breasts look like | ✓ | ✓ |
| How bodies function during sex (*sex can refer to any degree of sexual intimacy or behaviour) |  |  |
| How to sexually behave in order to keep my partner happy |  |  |
| About new things that turn me on |  |  |
| How genitals function during sex |  |  |
| About risks of physical harm associated with sex |  |  |
| What women should do during sex | ✓ |  |
| About sexual terminology |  |  |
| How to get sexual consent from my partner |  |  |
| About vaginal fisting |  | ✓ |
| About the scenarios where people have sex |  |  |
| What naked intersex persons’ bodies look like * (Intersex persons can have both male and female sex organs or other sexual characteristics) |  |  |
| About erogenous zones * (sensitive areas on the body that cause sexual arousal when they are touched) |  |  |
| About strap-on intercourse |  | ✓ |
| What genitals look like | ✓ | ✓ |
| How to ‘talk dirty’ |  | ✓ |
| About different sexual positions |  |  |
| How transgender persons behave during sex |  |  |
| How to sexually communicate using body language |  |  |
| About female orgasms |  | ✓ |
| About intersex person's orgasms |  |  |
| About oral sex on intersex genitals |  |  |
| Penetration of anus by finger(s) |  | ✓ |
| About anal sex |  | ✓ |
| About how to look hot in bed |  |  |
| Penetration of the vagina by finger(s) (fingering) |  |  |
| How normal my desires/fantasies are |  |  |
| About anal fisting |  | ✓ |
| How to please a sexual partner |  |  |
| About heterosexual sexual behaviours |  |  |
| About what is expected of a person when having sex |  | ✓ |
| My own sexual boundaries | ✓ |  |
| How to behave the way my sexual partner wants me to |  |  |
| How to communicate non-verbally during sex |  | ✓ |
| About vulva appearance (what the outside of the vagina looks like) |  | ✓ |
| How transgender people have sex |  |  |
| How to have sex |  |  |
| About the use of sex toys |  |  |
| Slang related to practices in same sex sexual activities |  |  |
| How to get your partner to finish quickly |  |  |
| About things I didn’t know would turn me on | ✓ |  |
| How to make someone orgasm |  |  |
| About masturbation |  |  |
| About your own sexual orientation (who you are attracted to) |  |  |
| How women behave during sex |  |  |
| About the use of condoms |  |  |
| What sexual behaviours non-binary people engage in *(Non-binary people do not identify as being male or female) |  |  |
| How to have group sex |  | ✓ |
| The sexual potential of the human body |  |  |
| How to interact with a partner during sex |  | ✓ |
| How to maintain my erection |  |  |
| How to have good sex |  |  |
| How to safely perform different sex behaviours |  |  |
| Attitudes surrounding condom use |  |  |
| How LGBT+ people are represented in porn |  |  |
| What to do if I feel physically threatened |  |  |
| Phrases related to LGBT+ people |  |  |
| About my gender identity (whether you identify as a man/woman/non-binary person) |  |  |
| About dominance and/or submission | ✓ |  |
| How to make a sexual partner have an orgasm |  | ✓ |
| What naked transgender persons’ genitals look like |  |  |
| About pubic/body hair |  |  |
| What sexual behaviours women engage in |  |  |
| How unusual my desires/fantasies are |  |  |
| How to act sexy in bed |  |  |
| About foreplay | ✓ |  |
| About bondage | ✓ |  |
| About penis size |  |  |
| How to maintain my partners erection |  |  |
| What sexual behaviours men engage in |  |  |
| How to fake an orgasm |  |  |
| About transgender people |  |  |
| The different roles people take during same sex encounters |  |  |
| Where things go during sex |  |  |
| How to ask for sexual consent from my partner |  |  |
| Why people have sex |  |  |
| How to do popular sexual behaviours |  |  |
| About the variety in body types |  |  |
| About role-playing | ✓ |  |
| How to maintain my partners vaginal lubrication |  |  |
| About the use of safe-words |  |  |
| About how long sex should last |  |  |
| About heterosexual sexual positions |  |  |
| How to get someone to have sex with you |  |  |
| About different fetishes |  |  |
| How to ‘turn on’ a sexual partner |  |  |
| How people interact with one another during sex |  |  |
| How to achieve mutual pleasure |  | ✓ |
| What naked intersex persons’ genitals look like |  |  |
| About vaginal Sex |  |  |
| How to masturbate |  |  |
| How to make a partner ‘squirt’ |  | ✓ |
| About anal rimming |  |  |
| How to give sexual consent to my partner |  |  |
| About sadomasochism | ✓ |  |
| What men should do during sex |  | ✓ |
| How to initiate sexual contact |  |  |
| What sexual behaviours transgender persons engage in |  |  |
| About heterosexual people |  |  |
| About same sex sexual behaviours |  |  |
| About oral sex on male genitals | ✓ |  |
| About what good sex is like |  | ✓ |
| How to be good in bed | ✓ | ✓ |
| How people with physical disabilities have sex |  |  |
| How to feel physically safe |  |  |
| How to read a sexual partners body language |  | ✓ |
| How to maintain Vaginal lubrication |  |  |
| About role playing |  |  |
| The emotional aspects of sex |  |  |
| About different sexual fantasies | ✓ |  |
